# Obeticholic acid ameliorates severity of Clostridioides difficile infection in high fat diet-induced obese mice

**DOI:** 10.1038/s41385-020-00338-7

**Published:** 2020-08-18

**Authors:** Shinsmon Jose, Anindita Mukherjee, Olivia Horrigan, Kenneth D. R. Setchell, Wujuan Zhang, Maria E. Moreno-Fernandez, Heidi Andersen, Divya Sharma, David B. Haslam, Senad Divanovic, Rajat Madan

**Affiliations:** 1 Division of Infectious Diseases, Department of Internal Medicine, University of Cincinnati College of Medicine, Cincinnati, OH 45220 USA; 2 Department of Pathology and Laboratory Medicine, Cincinnati Children’s Hospital Medical Center, Cincinnati, OH 45229 USA; 3 Department of Pediatrics, University of Cincinnati College of Medicine, Cincinnati, OH 45220 USA; 4 Division of Immunobiology, Cincinnati Children’s Hospital Medical Center, Cincinnati, OH 45229 USA; 5 Department of Pediatric Infectious Diseases, Cincinnati Children’s Hospital Medical Center, Cincinnati, OH 45229 USA; 6 Department of Pathology and Laboratory Medicine, University of Cincinnati College of Medicine, Cincinnati, OH 45220 USA; 7 Center for Inflammation and Tolerance, Cincinnati Children’s Hospital Medical Center, Cincinnati, OH 45229 USA; 8 Division of Gastroenterology, Hepatology and Nutrition, Cincinnati Children’s Hospital Medical Center, Cincinnati, OH 45229 USA; 9 Veterans Affairs Medical Center, Cincinnati, OH 45220 USA

## Abstract

Severe *Clostridiodes difficile* infection (CDI) is life-threatening and responds poorly to treatment. Obesity is associated with development of severe CDI. Therefore, to define the mechanisms that exacerbate disease severity, we examined CDI pathogenesis in high-fat diet (HFD)-fed obese mice. Compared to control mice, HFD-fed mice failed to clear *C. difficile* bacteria which resulted in protracted diarrhea, weight loss and colonic damage. After infection, HFD-induced obese mice had an intestinal bile acid (BA) pool that was dominated by primary BAs which are known promoters of *C. difficile* spore germination, and lacked secondary BAs that inhibit *C. difficile* growth. Concurrently, synthesis of primary BAs from liver was significantly increased in *C. difficile*-infected HFD-fed mice. A key pathway that regulates hepatic BA synthesis is via feedback inhibition from intestinal Farnesoid X receptors (FXRs). Our data reveal that the proportion of FXR agonist BAs to FXR antagonist BAs in the intestinal lumen was significantly reduced in HFD-fed mice after CDI. Treatment of HFD-fed mice with an FXR agonist Obeticholic acid, resulted in decreased primary BA synthesis, fewer *C. difficile* bacteria and better CDI outcomes. Thus, OCA treatment holds promise as a therapy for severe CDI.

## Introduction

*Clostridioides difficile* (*C. difficile*), the leading cause of nosocomial infections in the U.S., is responsible for >400,000 cases and 29,000 deaths every year.^1^ *C. difficile* is spread by fecal-oral transmission of bacterial spores.^2^ In the distal gastro-intestinal (GI) tract, *C. difficile* spores are converted to vegetative bacteria that produce toxins.^3^ *C. difficile* toxins damage the intestinal epithelium and elicit an intense inflammatory response, resulting in a diarrheal illness.^4^ Indigenous gut microbiota prevents *C. difficile* infection (CDI) by inhibiting spore germination and vegetative cell growth.^5^ An important biological mechanism by which gut commensals resist CDI is by modulating intestinal bile acid (BA) metabolome that in turn affects the bacterial lifecycle.^3,5–8^ Primary BAs [Cholic acid (CA) and chenodeoxycholic acid (CDCA)] are synthesized in the liver, conjugated with glycine or taurine, and released into the intestinal lumen where they are converted to unconjugated primary and secondary BAs by bacteria-derived enzymes.^9,10^ These BA metabolites directly influence various aspects of the *C. difficile* life cycle. In vitro, some primary BAs (e.g. CA; and its taurine-conjugated derivative, taurocholate, TCA) augment *C. difficile* spore germination to vegetative forms, whereas others (e.g. CDCA; alpha-muricholic acid, αMCA; beta-muricholic acid, βMCA) inhibit this process. On the other hand, most secondary BAs (e.g. deoxycholate, DCA; lithocholate, LCA; ursodeoxycholic acid, UDCA; hyodeoxycholate, HDCA; omega-muricholic acid, ωMCA) resist spore germination and arrest the growth of vegetative *C. difficile* bacteria.^7^ In vivo, the fecal BA profile of patients with recurrent and protracted CDI is dominated by primary BAs with fewer secondary BAs.^11^

The clinical disease spectrum of CDI ranges from asymptomatic colonization to mild, self-limited diarrhea to severe and fulminant colitis, which can cause death.^12^ Despite aggressive medical and surgical interventions, treatment of severe and fulminant CDI remains challenging and these patients continue to have a very high mortality.^13,14^ Published studies have shown that obesity is an independent predictor of severe CDI.^12,15,16^ To define the underlying mechanisms of severe CDI and uncover novel therapeutic targets, we have examined CDI pathogenesis in animal models of obesity. We previously studied CDI in mice that develop obesity as a result of mutations in leptin signaling pathway.^17^ To avoid the confounding effects of genetic deletion on host physiology,^18^ in this study we used a well-defined mouse model of obesity where wildtype (WT) C57BL/6 mice were fed an obesogenic high-fat diet (HFD) before infection.^19^ Here, we show that compared to RD (regular chow diet)-fed mice, HFD-induced obese mice exhibit severe and protracted CDI-induced diarrhea, weight loss, and colonic damage. In parallel, obese mice failed to adequately clear *C. difficile* bacteria. Persistence of *C. difficile* bacteria in colonic lumen of obese mice was associated with increased concentration of primary BAs that typically promote *C. difficile* germination and delayed recovery of secondary BAs that inhibit *C. difficile* growth. Our data further reveal that there was a decrease in the ratio of BAs that activate Farnesoid X receptor (FXR) to those that inhibit its feedback signaling to liver in the intestinal lumen of *C. difficile*-infected obese mice. Concurrently, hepatic synthesis of primary BAs was augmented in these mice. Suppressing the synthesis of primary BAs by administration of and FXR agonist, Obeticholic acid (OCA) decreased *C. difficile* bacterial burden and ameliorated clinical disease in obese mice. Since OCA is already FDA-approved for human use,^20^ it represents a novel therapeutic approach to reducing CDI severity in an obese host.

## Results

### HFD-induced obese mice exhibit severe, protracted CDI

**Fig. 1:**
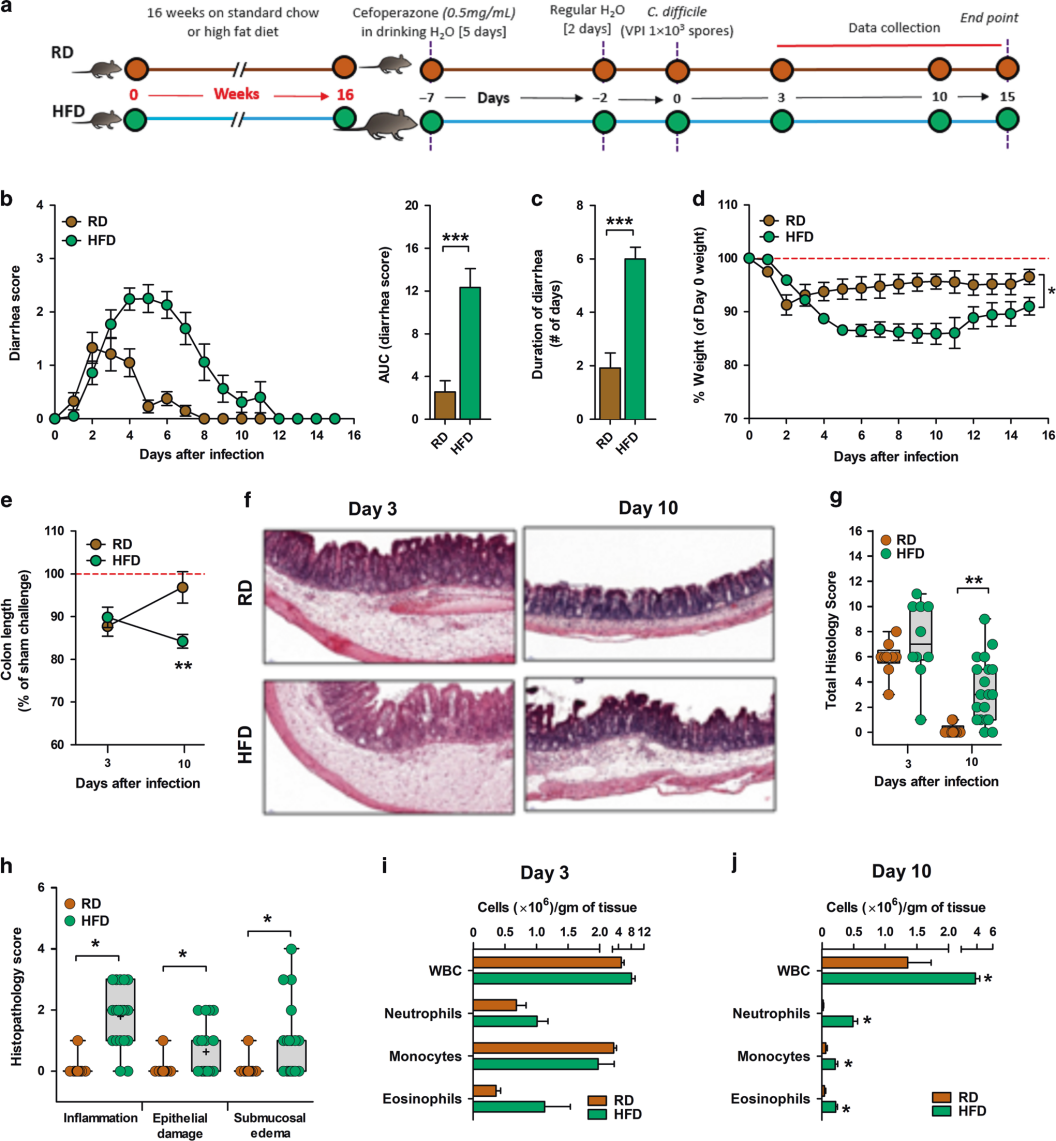
HFD-induced obese mice have protracted C. difficile infection (CDI). Experimental plan: RD-fed and HFD-fed mice were pre-treated with antibiotics for 5 days in drinking water and challenged with 1 × 103 C. difficile (VPI 10463) spores by oro-gastric gavage two days after cessation of antibiotics. Animals were monitored until day 15, scored for clinical symptoms, and sacrificed at day 3 and 10 to collect samples (**a**). Disease severity was evaluated based on diarrhea score (**b**), duration of diarrhea (**c**), and percentage change in body weight after CDI (compared to day 0 weight) (**d**). Extent of tissue damage was evaluated based on changes in colon length (**e**), and histological findings (**f**–**h**). Representative H&E images of cecal sections (f) and total histology score (**g**) from day 3 and day 10 of CDI. Scores of individual histological parameters (inflammation, epithelial damage, and submucosal edema) on day 10 of CDI (**h**). Number of WBCs, neutrophils, monocytes and eosinophils in cecal tissue (**i**–**j**) on day 3 (**i**) and day 10 (**j**) of CDI. Data are means ± SEM. *n* = 10–21 for **b**–**d**; *n* = 10–18 for **e**, **g**, **h**; *n* = 6–10 for **i**, **j**; data presented in **b**–**e** and **g**, **h** pooled from 2 to 3 experiments. *p < 0.05, **p < 0.01, ***p < 0.001; repeated measures ANOVA (**b**, **d**), and two-tailed Student’s *t*-test (**c**, **e**, **g**–**j**).

Six-to-eight-week-old C57Bl/6 male mice were fed HFD or RD *ad libitum* for a period of 14–16 weeks (Supplementary Fig. 1a). With consistent high-calorie uptake, HFD-fed mice exhibited significant weight gain and developed metabolic sequelae of obesity including impaired glucose tolerance (Supplementary Fig. 1b–d). As reported previously, naïve HFD-fed mice had more total leukocytes in lamina propria, but the number of neutrophils, monocytes, and eosinophils were similar to RD-fed mice (Supplementary Fig. 1e).^21^ After induction of obesity, mice were pre-treated with antibiotics and infected with *C. difficile* spores, as per our previously published protocol (Fig. 1a).^22^ All mice developed diarrhea and exhibited weight loss (Fig. 1b, d) within first two days of infection, but peak diarrhea score was higher in HFD-fed mice and they had protracted disease (Fig. 1b, c). Despite remaining on a high-calorie diet, HFD-fed mice also exhibited prolonged weight loss (Fig. 1d). Although uninfected HFD-fed mice had shorter colons compared to RD-fed mice (Supplementary Fig. 1f), microscopic examination of H&E stained cecal sections did not reveal any evidence of submucosal edema, epithelial damage, and inflammatory cell infiltration in either group (Supplementary Fig. 1g, h). During acute CDI (day 3 after infection), significant tissue damage was observed in both groups of mice, as evidenced by colon length shortening and histology scoring of cecal tissue (Fig. 1e–h). However, while tissue damage in RD-fed mice improved by day 10 after infection, colonic injury persisted in HFD-fed mice (Fig. 1e–h). Similarly, the number of infiltrating neutrophils, monocytes, and eosinophils that increased in both groups of mice during acute CDI, remained significantly elevated in HFD-fed mice till day 10 after infection (Fig. 1i–j). Taken together these data indicate that persistence of colonic innate immune cells and tissue injury observed in HFD-fed mice in our model is a result of CDI.

### HFD-induced obese mice have delayed C. difficile clearance

**Fig. 2:**
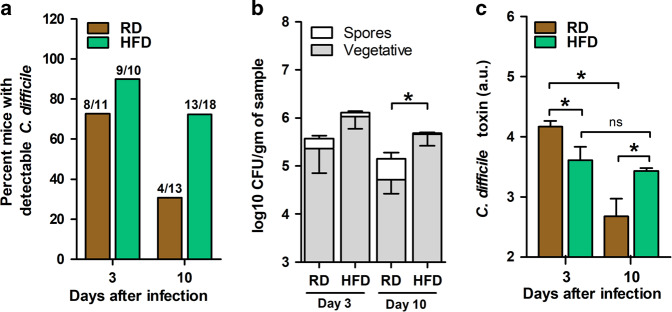
HFD-induced obese mice exhibit delayed C. difficile clearance. Proportion of mice with detectable C. difficile on days 3 and 10 of CDI; Fraction on top of each bar indicates the # of mice with detectable C. difficile over total # of mice challenged with C. difficile (**a**). C. difficile spores, vegetative bacteria (**b**) and toxin A and B levels (**c**) in cecal contents of RD-fed and HFD-fed mice. **b**, **c** Samples with detectable C. difficile on day 3 and day 10 after infection were used. Data are means ± SEM. *n* = 10–18 for a–b; *n* = 7–9 for **c**; pooled from 2 to 3 experiments. *p < 0.05, n.s. non-significant; two-tailed Student’s *t*-test.

**Fig. 3:**
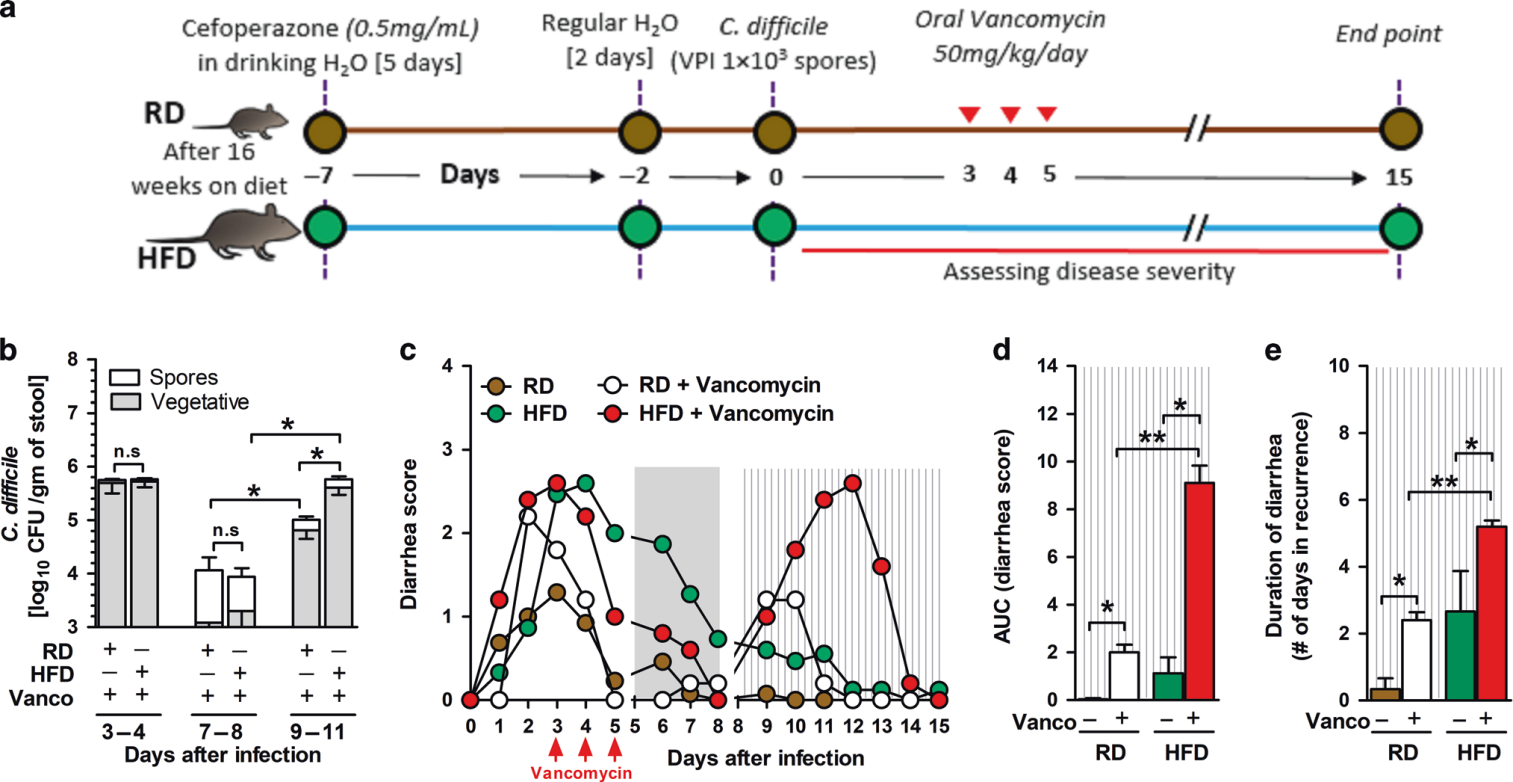
Reduction of C. difficile pathogen burden by vancomycin treatment alleviates diarrhea. Experimental plan: RD fed and HFD-fed mice were pre-treated with antibiotics for 5 days in drinking water and challenged with 1 × 103 C. difficile spores. Animals were treated with 50mg/kg/day vancomycin on days 3, 4, and 5 by oral gavage and monitored for clinical symptoms (**a**). C. difficile CFU in stool samples of RDfed and HFD-fed mice that were treated with vancomycin (**b**) C. difficile CFU in untreated control groups are presented in Supplementary Fig. 2b. Disease severity was evaluated based on diarrhea score (**c**, **d**) and duration of diarrhea (**e**). Gray shaded area represents effect of vancomycin treatment, and vertical lines in the background represent recurrent CDI. (Data are means ± SEM. *n* = 7–8 pooled from 2 experiments. *p < 0.05; **p < 0.01, n.s. non-significant; two-tailed Student’s *t*-test.

On day 3 after infection, majority of mice had detectable *C. difficile* (spores + vegetative forms) in cecal contents (Fig. 2a), and most of the bacteria were in vegetative state (Fig. 2b). By day 10, most RD-fed mice had cleared *C. difficile*, but >70% HFD-fed mice still had detectable *C. difficile* in cecal contents (Fig. 2a), and these mice also had significantly more vegetative *C. difficile* forms (Fig. 2b). Vegetative *C. difficile* forms produce the toxins that are responsible for tissue damage and clinical disease.^4^ Similar to the dynamics of vegetative *C. difficile* bacteria, RD-fed mice had a significant decline in toxin levels by day 10 after infection, whereas toxin titers remained elevated in HFD-fed mice (Fig. 2c). Vancomycin is a mainstay of CDI therapy, and kills vegetative *C. difficile* without affecting spores.^13^ To study the role of vegetative *C. difficile* in regulating disease severity, we treated mice with oral vancomycin for 3 days at the peak of diarrhea (Fig. 3a). Vancomycin treated mice had significant decrease in vegetative *C. difficile* on days 7–8 after infection with a concurrent reduction in diarrhea (Fig. 3b-c and Supplementary Fig. 2a). Of note, vancomycin can disrupt normal gut microbiota to create a colonic micro-environment that is favorable for *C. difficile* growth.^8^ Indeed, 4 days after stopping vancomycin (i.e. between days 9 and 11 after initial infection), *C. difficile* growth rebounded and diarrhea resumed (Fig. 3b–e). Similar to the initial episode, HFD-fed mice exhibited more severe recurrent CDI (Fig. 3c–e and Supplementary Fig. 2b). Together, these data indicate that vegetative *C. difficile* persistence is a key driver of disease severity in HFD-fed obese mice.

### HFD-induced obese mice have delayed recolonization of gut microbiota

**Fig. 4:**
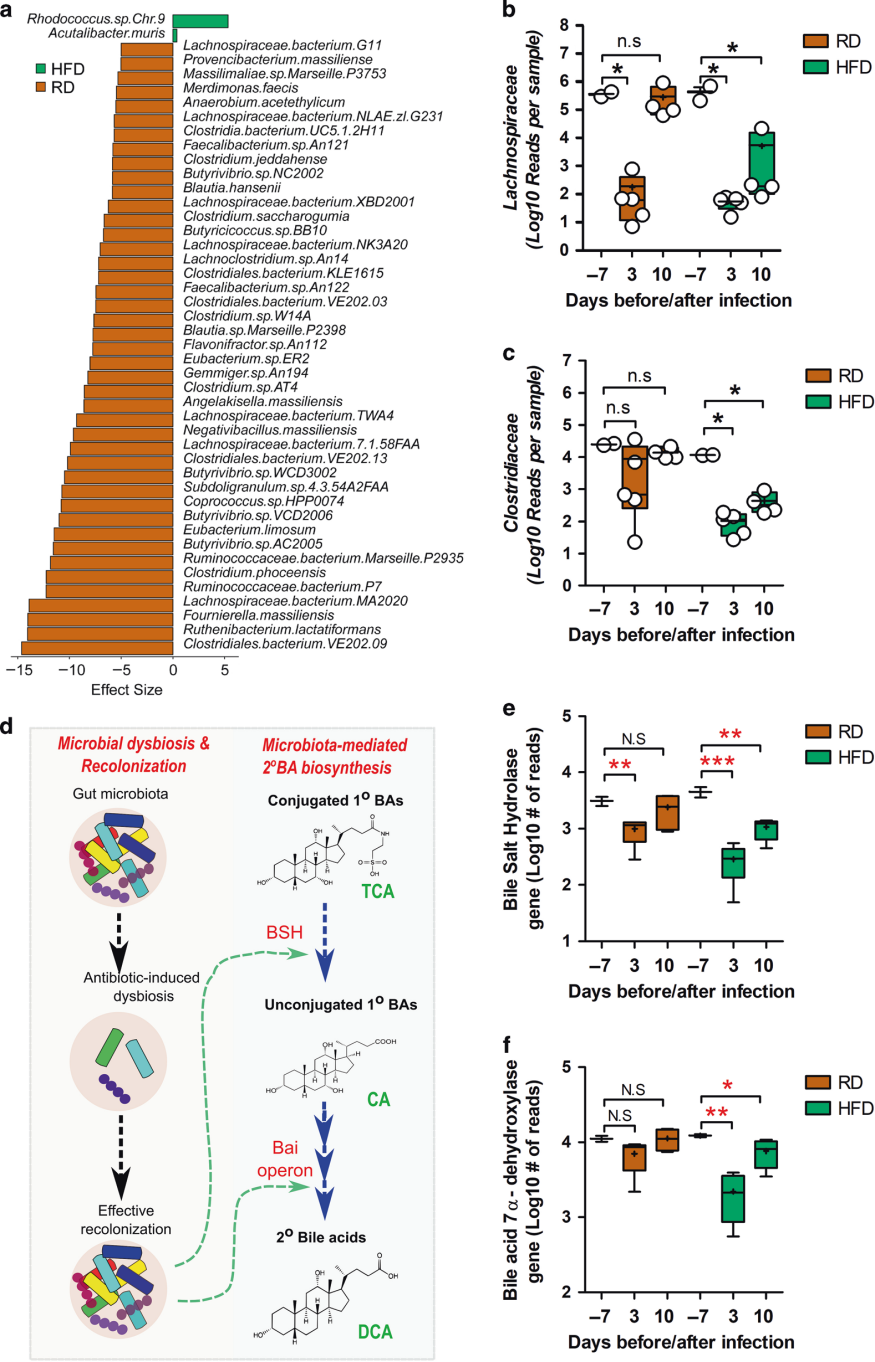
Restoration of gut microbiota and restitution of microbial enzymes is delayed in obese mice. Shrinkage linear discriminant analysis of species that significantly affect the microbial community on day 10 of CDI (Cut off *p* value set at 0.025 and effect size at 0.3) (**a**). Abundance of Lachnospiraceae (**b**) and Clostridiaceae (**c**) in cecal contents. Graphic representation of influence of microbial recolonization on BA metabolism (**d**). Number of reads of bile salt hydrolase (**e**) and Bile acid 7-α dehydroxylase (**f**) genes in cecal contents. Data are means ± SEM. *n* = 2–5. *p < 0.05; **p < 0.01; ***p < 0.001, n.s. non-significant; two-tailed Student’s *t*-test.

Since normal gut microbiota resists *C. difficile*, we posited that delayed recovery of gut commensals in HFD-fed mice could result in pathogen persistence. To define changes in gut microbiome, we performed metagenomic sequencing on cecal contents from RD-fed and HFD-fed mice prior to and after CDI. Principal component analyses and estimation of overall bacterial richness revealed that on day 3 after infection, gut microbiota of RD-fed and HFD-fed mice was significantly different, compared to respective naïve control mice (Supplementary Fig. 3a). By day 10 after infection, gut microbiota of RD-fed mice had recovered and was similar to naïve mice, but that of HFD-fed mice was very similar to mice with acute CDI (Supplementary Fig. 3a, b). To identify differences at the species level, we performed shrinkage linear discriminant analysis and compared the abundance of bacterial strains that were most different between *C. difficile*-infected RD-fed and HFD-fed mice on day 3 (Supplementary Fig. 3c) and day 10 (Fig. 4a) after infection. At day 10 after infection, a total of 45 bacterial strains had a large effect size that discriminated RD-fed mice from HFD-fed mice (Unadjusted *p* value < 0.025 and effect size > 0.3; Fig. 4a), and 18 of these belonged to either *Lachnospiraceae* or *Clostridiaceae* families. Importantly, the bacterial strains assigned to these families were similar to pre-infection levels in RD-fed mice, but their abundance remained low in HFD-fed mice (Fig. 4b, c). Since *Lachnospiraceae* and *Clostridiaceae* are associated with CDI resistance in humans and in animal models,^8,23,24^ our data indicate that lack of recolonization of these particular commensals may contribute to *C. difficile* persistence in obese mice.

Gut commensals affect colonic BA composition by enzyme-mediated transformation of liver-derived primary BAs: bacteria-derived bile salt hydrolases (BSHs) and 7α-dehydroxylases convert conjugated primary BAs to unconjugated BAs and to secondary BAs, respectively (Fig. 4d). BSHs are expressed by bacteria belonging to both *Lachnospiraceae* and *Clostridiaceae* families.^25^ Although the operon that encodes for 7α-dehydroxylase called bile acid inducible (*bai*) is present in very few gut microbes, most of those belong to *Clostridiaceae* family.^25^ Consistent with reduction in *Lachnospiraceae* and *Clostridiaceae* families, expression of both *bsh* and *7α-dehydroxylase* declined in cecal contents of all mice during acute CDI. Similar to recovery of gut microbiota, by day 10 these enzymes reverted to pre-infection levels in RD-fed mice but their expression remained low in HFD-fed mice (Fig. 4e, f). Together, these data demonstrate a lack of appropriate bacterial recolonization and enzymatic restitution in *C. difficile*-infected HFD-fed mice, which could create a colonic BA profile that promotes pathogen persistence.

### Cecal BA pool is perturbed for a longer duration in HFD-induced obese mice after CDI

**Fig. 5:**
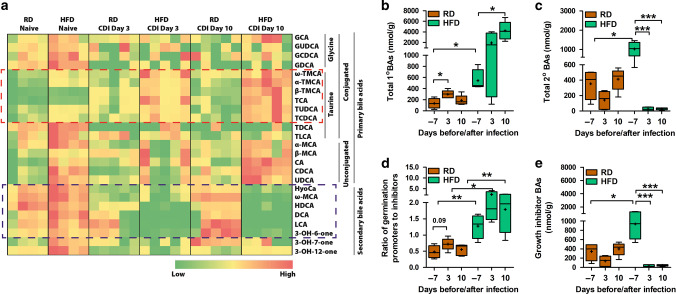
CDI alters BA homeostasis in HFD-fed mice. Relative amounts of BA metabolites in cecal contents of RD-fed and HFD-fed mice are displayed as a heat map. Each sub-column represent BA profile of individual mice (**a**). Concentration of primary BAs (**b**) and secondary BAs (**c**). Ratio of BAs that are C. difficile spore germinants (TCA and CA) to those that are germination inhibitors (CDCA, UDCA, αMCA, βMCA, ωMCA, HDCA, LCA and DCA) (**d**) concentration of BAs that inhibit C. difficile growth (CDCA, UDCA, ωMCA, HDCA, LCA, and DCA) (**e**) in cecal contents. Data are means ± SEM. *n* = 4–5. *p < 0.05; **p < 0.01, ***p < 0.001, n.s. non-significant; two-tailed Student’s *t*-test.

Liver-derived primary BAs and microbiota-derived secondary BAs impact *C. difficile* lifecycle.^7,8,25^ Specifically, conjugated primary BAs promote bacterial spore germination, whereas secondary BAs inhibit *C. difficile* germination and vegetative cell growth.^7,25^ We quantified cecal BA metabolites before and after CDI by liquid-chromatography tandem mass spectrometry (LC-MS). Congruent with published studies that diet and obesity affect BA metabolism and their composition,^26,27^ we found significant differences between cecal BA profiles of uninfected mice (Fig. 5a, left 2 panels): HFD-fed mice had higher levels of both primary and secondary BAs compared to RD-fed mice (Fig. 5b, c and Supplementary Fig. 4). Antibiotic pre-treatment followed by CDI altered the cecal BA composition in both groups, and during acute infection the quantity of total primary BAs was significantly increased and secondary BAs were reduced in all mice (Fig. 5a–c and Supplementary Fig. 4). By day 10 after infection, amounts of primary and secondary BAs reverted to pre-infection levels in RD-fed mice, but cecal BA profiles of HFD-fed mice remained altered (Fig. 5a–c and Supplementary Fig. 4). In fact, concentration of conjugated primary BAs which typically promote *C. difficile* germination, continued to increase in HFD-fed mice and these BAs were >100-fold higher at day 10 after infection, compared to uninfected control mice (Supplementary Fig. 4). On the other hand, secondary BAs were almost completely ablated in HFD-fed mice during acute CDI, and these did not return to pre-infection levels till at least day 10 after infection (Fig. 5c and Supplementary Fig. 4).

Categorization of these BA metabolites based on their known effects on *C. difficile* life cycle revealed that prior to infection, the ratio of *C. difficile* spore germinant to inhibitor BAs was increased in HFD-fed mice (Fig. 5d). Interestingly, the concentration of BAs that inhibit vegetative *C. difficile* growth was also higher in HFD-fed mice (Fig. 5e). As would be expected, cecal contents of uninfected HFD-fed mice promoted *C. difficile* spore germination but not vegetative cell growth, whereas cecal contents from uninfected RD-fed mice did not permit either germination or vegetative cell growth (Supplementary Fig. 5a–c). Consistent with high vegetative *C. difficile* burden during acute CDI (Fig. 2b), the relative proportion of *C. difficile* spore germinant BAs were increased in all mice (Fig. 5d). However, while this ratio reverted back to pre-infection levels in RD-fed mice by day 10 after infection, they were persistently elevated in HFD-fed mice (Fig. 5d). In addition, concentration of BA metabolites that inhibit vegetative *C. difficile* growth dropped dramatically in HFD-fed mice during acute CDI and did not recover even by day 10 of infection (Fig. 5e).

### CDI augments primary BA synthesis in HFD-induced obese mice

**Fig. 6:**
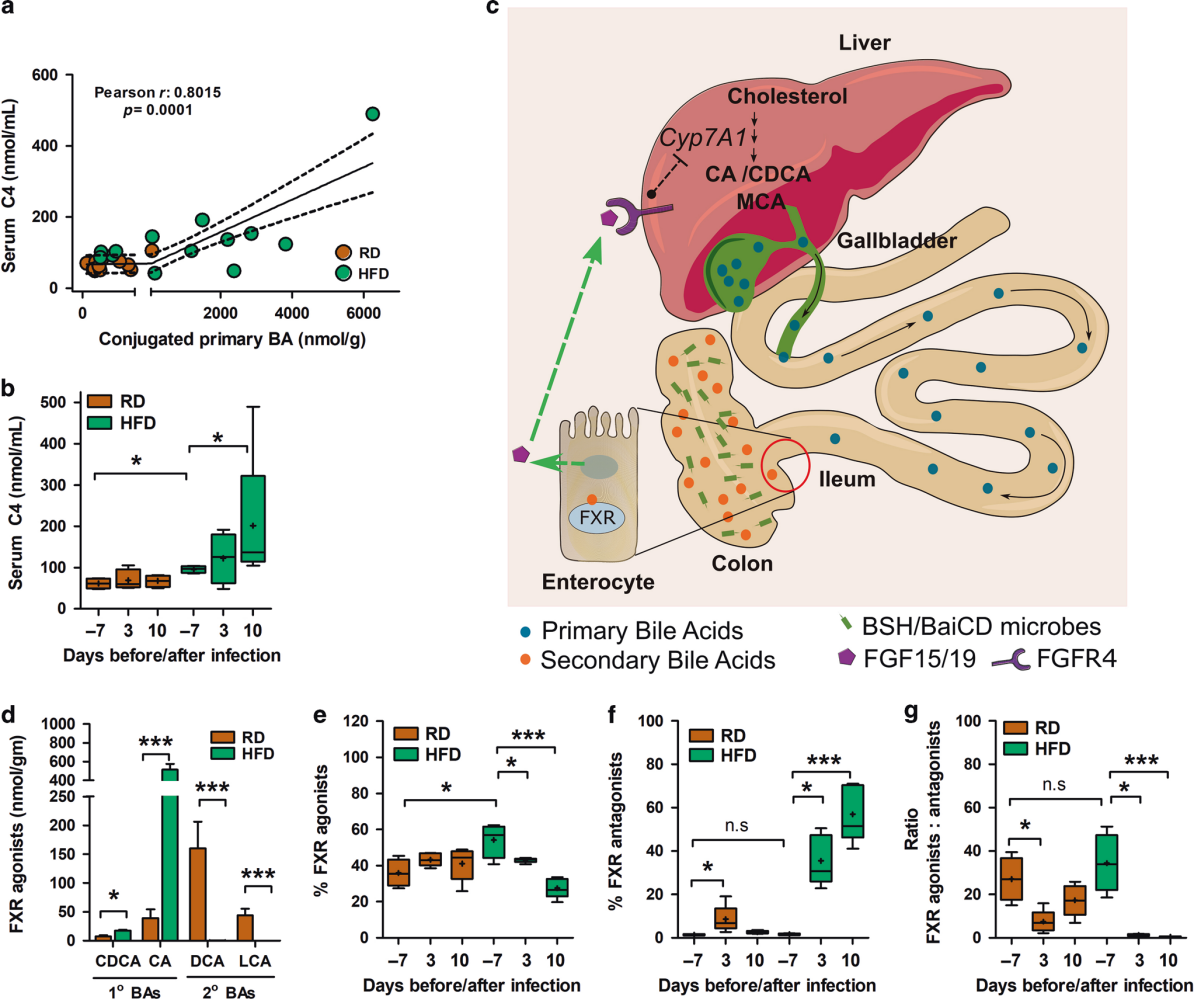
CDI alters concentration of cecal FXR ligands in HFD-fed mice and increases primary BA synthesis. Pearson correlation of serum C4 and conjugated primary bile acids in cecal contents (**a**). Serum C4 levels in RD-fed and HFD-fed mice (**b**). Schematic representation of hepatic BA synthesis feedback regulation by FXR signaling. BAs that are FXR agonists trigger the transcription and release of fibroblast growth factors (FGFs) 15/19 that inhibit Cyp7A1, the rate-limiting enzyme in hepatic BA synthesis (**c**). Concentration of CDCA, CA, DCA, and LCA in cecal contents (**d**). Percentage of BAs that are FXR agonists (**e**), antagonists (**f**) and ratio of FXR agonists to antagonist (**g**) in cecal contents. Data are means ± SEM. *n* = 28 for **a**; *n* = 4–5 per group for **b**, d–g. *p < 0.05, **p < 0.01, ***p < 0.001, n.s. non-significant; two-tailed Student’s *t-*test.

Although lack of bacteria-derived enzymes and the consequent decreased deconjugation could result in excess conjugated primary BAs in HFD-fed mice, the sustained nature of this increase suggested an added contribution from BA bio-synthetic machinery. To examine BA synthesis, we measured 7α-hydroxy-4-cholesten-3-one (or C4).^28^ C4 mirrors the activity of a rate-limiting enzyme in BA synthesis pathway (Cyp7a1) and is secreted from liver during conversion of cholesterol to BAs.^28^ Notably, serum C4 concentration is a highly specific marker of primary BA synthesis.^29^ Prior to infection, serum C4 was slightly more in HFD-fed mice compared to RD-fed mice, suggesting a role for dietary modifications in inducing hepatic BA synthesis. After CDI, we found a strong correlation between serum C4 concentration and amounts of conjugated and total primary BAs in cecal contents (Fig. 6a and Supplementary Fig. 4c). Further, while serum C4 level did not change after infection in RD-fed mice, it significantly increased in *C. difficile*-infected HFD-fed mice (Fig. 6b).

An important mechanism that regulates hepatic BA synthesis is by feedback signaling from intestinal Farnesoid X receptor (FXR) pathway.^30^ FXR agonists trigger the transcription and release of fibroblast growth factor (FGF) 15/19 from intestinal epithelial cells while FXR antagonists reduce FGF 15/19 expression.^30^ FGF 15/19 in turn inhibit hepatic BA synthesis by blocking Cyp7a1 expression (Fig. 6c).^30^ Many natural BAs activate FXR signaling in a concentration-dependent manner.^31,32^ Among these, CDCA is the most potent FXR activator, followed by DCA and LCA, while CA is the least potent FXR agonist.^31,32^ We observed that compared to RD-fed mice, the levels of DCA and LCA (i.e. FXR agonists with moderate potency) declined dramatically in HFD-fed mice and these BAs were 500–1500 fold lower on day 10 after CDI (Fig. 6d and Supplementary Fig. 4a). Although the concentration of CDCA and CA (both high potency and low potency FXR agonists) increased in HFD-fed mice on day 10 after infection, the proportion of this increase was not as large and it ranged from 2–13-fold, compared to RD-fed mice (Fig. 6d and Supplementary Fig. 4a). Concurrently, HFD-fed mice had a substantial increase in BAs that are potent FXR antagonists (UDCA, GUDCA α-TMCA, and β-TMCA; Fig. 6f and Supplementary Fig. 4a, b).^33,34^ Altogether, while the ratio of total FXR agonists to FXR antagonists decreased in both groups during acute CDI, the degree of decline was higher in HFD-fed mice and this ratio did not revert back to pre-infection levels in these mice (Fig. 6g). These data support a scenario whereby reduced feedback signaling from intestinal FXR pathway could contribute to the increase in hepatic BA synthesis seen on day 10 after CDI in HFD-fed mice.

### Obeticholic acid alleviates CDI in HFD-induced obese mice

**Fig. 7:**
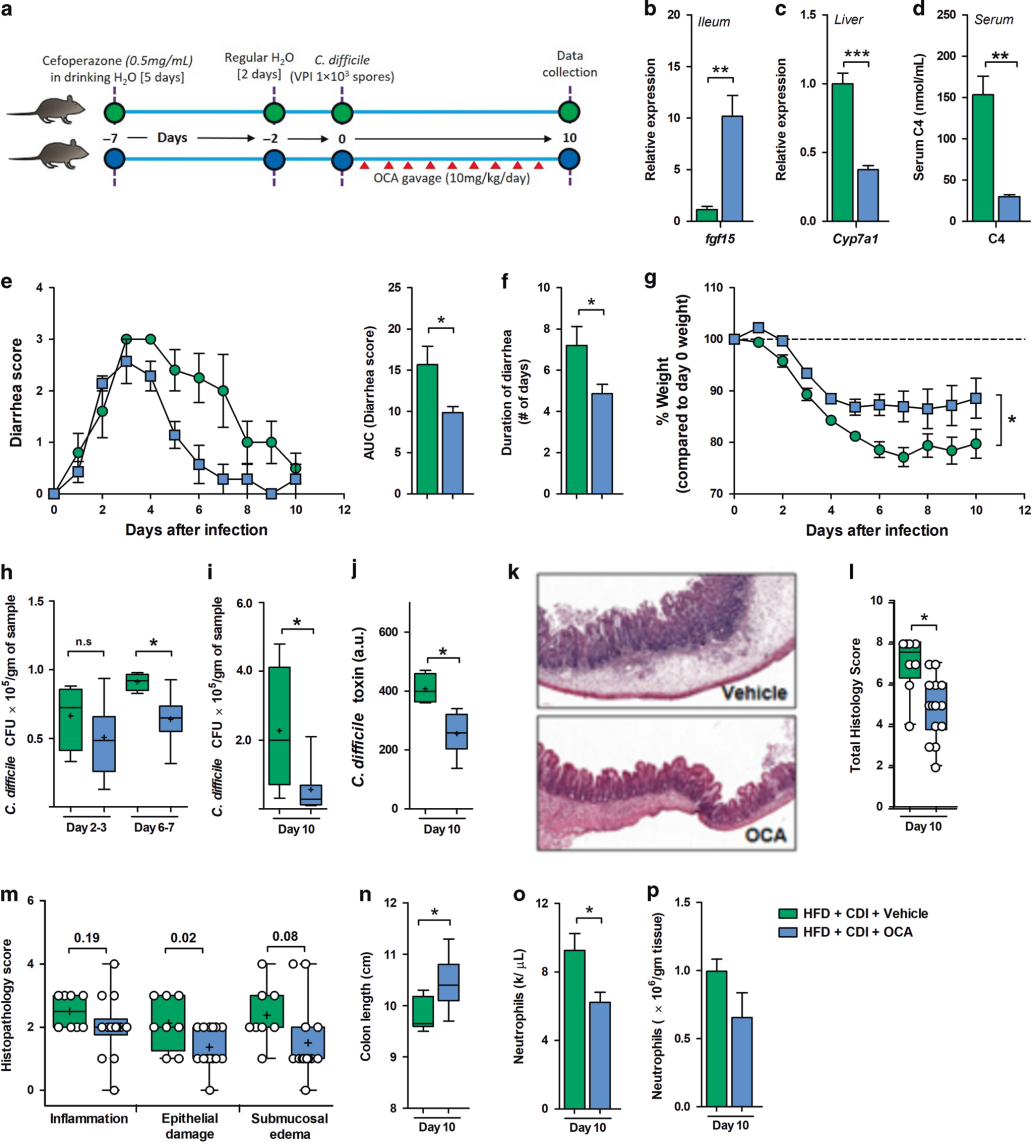
Obeticholic acid activates FXR signaling, reduces C. difficile burden and ameliorates CDI. Experimental plan: HFD-fed mice were pre-treated with antibiotics and challenged with 1×103 C. difficile (VPI 10463) spores. Animals were gavaged with 10 mg/kg/day OCA or vehicle every 24 h and monitored daily for clinical symptoms (**a**). mRNA expression of fgf15 in distal ileum (**b**), Cyp7a1 in liver tissue (**c**), and C4 concentration in serum (**d**) on day 10 of CDI. Disease severity was evaluated based on diarrhea score (**e**), duration of diarrhea (**f**), and percent body weight change after CDI (**g**). C. difficile burden in stool samples (**h**), cecal contents (**i**), and C. difficile toxin A/B levels in cecal contents (**j**). Extent of tissue damage was evaluated based on changes in histological findings (**k**–**m**) and colon length (**n**) on day 10 after infection. Number of neutrophils in peripheral blood (**o**) and cecal tissue (**p**) on day 10 after infection. Data are means ± SEM. *n* = 6 for **b**–**d**; *n* = 9–14 for **e**–**n**; *n* = 6 for **o** and **p**; data presented in **e**–**n** are pooled from 2 independent experiments. *p < 0.05, **p < 0.01, ***p < 0.001; two-tailed Student’s t-test.

Obeticholic acid (OCA) is a highly selective FXR agonist that is FDA-approved for treatment of primary biliary cholangitis.^20^ OCA inhibits primary BA synthesis by activating intestinal FXR signaling.^20,35^ To examine its role as a potential therapy for CDI, we treated *C. difficile*-infected mice daily with OCA, starting 12 h after infection (Fig. 7a). Clinical disease severity was assessed every day and tissue samples were collected after 10 days of therapy. As expected, OCA treatment-induced *fgf15* expression in terminal ileum, decreased hepatic *Cyp7a1* expression, and reduced serum C4 levels in both RD-fed and HFD-fed mice (Fig. 7b–d and Supplementary Fig. 6a–c). However, OCA treatment reduced diarrhea duration and severity as well as CDI-induced weight loss only in HFD-fed mice (Fig. 7e–g) without affecting CDI severity in RD-fed mice (Supplementary Fig. 6d–g). Notably, OCA treatment did not affect acute CDI in either group, but ameliorated disease during later phases in HFD-fed mice (Fig. 7e, g). Mirroring the changes in disease severity, OCA treated HFD-fed mice had fewer *C. difficile* bacteria in stool and in cecal contents during the later phases of infection (Fig. 7h, i). Toxin titers were also lower in cecal contents of OCA-treated HFD-fed mice on day 10 after infection (Fig. 7j). Consistent with reduced pathogen, OCA-treated HFD-fed mice had significantly less colonic injury on day 10 after infection, as evidenced by reduced epithelial damage, improvement in overall histopathology and colon length (Fig. 7k–n). Although OCA-treated HFD-fed mice had fewer blood neutrophils, the number of tissue neutrophils were similar to untreated mice (Fig. 7o, p). Together our data show that, OCA treatment significantly reduced hepatic BA synthesis in all mice. However, the effects on *C. difficile* clearance and CDI resolution were observed only in the case of HFD-fed mice who exhibit protracted disease, whereas RD-fed mice followed their usual pattern of spontaneous pathogen clearance and disease resolution.

## Discussion

Severe CDI is an important clinical problem and obesity is a risk factor that predicts severe disease.^12,15,16^ To investigate the mechanisms that exacerbate CDI severity, we therefore coupled a well-established model of diet-induced obesity to an animal model of CDI. Using this model, we show that HFD-induced obese mice: (i) failed to clear *C. difficile* bacteria and exhibited protracted diarrhea and tissue injury; (ii) had delayed restoration of gut microbiome and BA metabolome after CDI; and (iii) exhibited increased primary BA synthesis after CDI. Finally, OCA treatment reduced primary BA synthesis, expedited *C. difficile* clearance and resulted in faster CDI resolution in this group of mice.

Although effects of BAs on CDI pathogenesis have been previously examined,^6–8,25^ this is the first study to comprehensively examine the role of obesity-associated changes in gut BA metabolome in regulating CDI severity. It has been previously reported that cefoperazone-mediated disruption of gut microbiota in non-obese mice results in a dominance of primary BAs and lack of secondary BAs leading to a colonic milieu that promotes *C. difficile* germination.^8^ We show that cefoperazone and CDI in obese mice result in a cecal BA pool dominated by promoters of spore germination and a loss of BAs that inhibit its growth. Importantly, our data reveal that these intestinal BA derangements lasted much longer in HFD-fed mice: these mice had high concentration of primary BAs and an almost complete depletion of secondary BAs in GI lumen by day 3 of CDI and recovery of BA profile to pre-infection level did not happen till day 10 after infection. In addition, our novel data reveal that HFD-fed mice exhibited a substantial and sustained increase in hepatic synthesis of primary BAs after infection. The increased availability of primary BAs and concomitant decrease in secondary BAs could therefore support pathogen persistence in obese mice. Since exogenous feeding of primary BAs (e.g. CA) can reduce the abundance of gut bacteria including those that are capable of 7α dehydroxylation,^36–38^ the excess primary BA synthesis and their release into GI tract in a *C. difficile*-infected obese host could prevent gut microbiota recolonization and create a niche that supports *C. difficile* persistence, resulting in prolonged diarrhea. In contrast, there was no effect of CDI on primary BA synthesis in RD-fed mice, which could allow for faster restoration of gut microbiota to pre-infection levels. In these mice, early restoration of microbiota and BA metabolizing enzymes was associated with improved control of *C. difficile* (Fig. 4 and Supplementary Fig. 3a, b).

FXR signaling from GI tract is an important mechanism that regulates synthesis of primary BAs by liver.^30^ In our experiments, the increased hepatic primary BA synthesis (i.e. serum C4 amplification) was observed only in HFD-fed mice, whereas RD-fed mice had no changes in serum C4 levels (Fig. 6b). Similar to changes in serum C4, the decline in FXR agonists was observed only in HFD-fed mice after CDI, whereas RD-fed mice had no changes in proportion of FXR agonists (Fig. 6e). Thus, an altered balance between FXR agonists and antagonists in obesity could reduce feedback inhibition of hepatic BA synthesis. OCA is a selective FXR agonist,^20,35^ and OCA treated HFD-fed mice exhibited an increase in expression of molecules that activate FXR signaling (Fig. 7b). Concurrently, there was reduction in hepatic BA synthesis, faster *C. difficile* clearance and clinical disease resolution in this group. Although the role of FXR signaling in CDI pathogenesis is not completely understood, diarrhea resolution in patients with recurrent CDI was associated with activation of FXR signaling pathways.^39^ We observed that 10 days of OCA treatment-induced FXR signaling molecules and reduced hepatic BA synthesis in RD-fed mice as well, but this change did not affect overall CDI severity in this group. A possible explanation is that RD-fed mice recover their gut microbiome and BA metabolome rapidly and therefore the effects of FXR agonism on CDI remain undetectable in this group. Notably, data from OCA-treated HFD-fed mice supports this idea since acute CDI remained unaffected even in HFD-fed mice. Together our studies and published literature suggest that FXR-mediated hepatic primary BAs synthesis may play an important role in CDI pathogenesis, and provide a strong rationale for a thorough examination of FXR signaling in this disease. Future studies employing FXR-deficient mice and alternative FXR agonists to elucidate the timing and duration of FXR activation after CDI in obese and non-obese mice are clearly warranted.

Although our data suggests that OCA acts via changing hepatic BA synthesis, it remains a possibility that OCA, which is a BA analog has direct effects on *C. difficile* growth. Such effects of OCA have been reported in vitro when tested at a concentration of 215 µM.^40^ However, whether such concentrations are achieved in vivo remains to be elucidated. Our current study has also not defined whether the driver of CDI severity is diet or obesity-associated colonic inflammation or a combination of these factors. Diet-associated perturbation of *Lachnospiraceae* after antibiotic treatment and similar reduction in resistance to CDI has been previously reported.^41,42^ In addition, the duration of dietary modifications used to induce obesity in our studies does result in low-grade colonic inflammation. Of particular interest in CDI pathogenesis is the role of colonic neutrophilia. Studies have clearly shown that exaggerated colonic neutrophilia is associated with increased tissue damage, severe clinical disease and high mortality.^43–45^ Our data show that while the number of colonic neutrophils were similar in RD-fed and HFD-fed mice prior to infection (Supplementary Fig. 1e) and both groups had similar intensity of colonic neutrophilia during acute CDI (Fig. 1i), but obese mice had longer duration of neutrophilia (Fig. 1j). These data suggest that pathogen persistence is likely the main driver of prolonged tissue neutrophilia in an obese host. However, pathogen persistence despite significant and prolonged neutrophil infiltration is also counterintuitive. Whether obesity-associated sequelae adversely affect anti-microbial functions of neutrophils remains to be explored.

CDI is typically a nosocomial infection, however a robust increase in the incidence of community-onset CDI (CO-CDI) has been recently reported.^46,47^ Our data shows that cecal contents from HFD-induced obese mice without any antibiotic exposure promote *C. difficile* spore germination (Supplementary Fig. 5b). Importantly, these findings suggest that the obesity epidemic could contribute to increasing cases of CO-CDI. However, despite presence of vegetative *C. difficile* after exposure to *C. difficile* spores, obese mice had only a partial disease: they exhibited significant weight loss (Supplementary Fig. 5d) but did not develop diarrhea unless they were pre-treated with cefoperazone (data not shown). This finding raises the possibility of a threshold effect for dysbiosis that is permissive for *C. difficile* colonization and growth. In the setting of obesity (without antibiotics), gut microbiome dysbiosis and altered BA metabolome may be enough to promote *C. difficile* germination and initial colonization, but insufficient to generate a critical mass of toxin-producing vegetative *C. difficile* to cause diarrhea. A second hit (e.g. by using antibiotics) could then cause significant disruption of microbiota and BA metabolome to promote vegetative *C. difficile* growth and toxin production leading to severe disease. Indeed, despite challenge with a 10-fold lower inoculum of *C. difficile* spores (10^3^/mouse in cefoperazone-treated mice vs 10^4^/mouse without antibiotic pre-treatment), the vegetative *C. difficile* CFUs were ~20-fold higher in obese mice that received cefoperazone and had significant dysbiosis (Fig. 2b and Supplementary Fig. 5e).

In sum, we have developed a new model of severe CDI by using HFD-induced obese mice. Using this model, we show that HFD-induced obesity fundamentally alters the entero-hepatic BA metabolism pathway to create a niche that favors *C. difficile* germination and growth. Our data support a scenario in which microbial dysbiosis and lack of efficient recolonization in obese mice alters BA metabolism to worsen CDI. Treatment of obese mice with OCA reduced *C. difficile* burden and improved CDI suggesting that targeting this pathway has potential translational value in CDI therapeutics.

## Materials and methods

### Mice

All mice were maintained at the animal facility in University of Cincinnati, accredited by the American Association for Accreditation of Laboratory Animal Care (Frederick, MD), under protocols approved by the Institutional Animal Use and Care Committee. 6–8 week old male C57BL/6 J mice (Jackson Laboratories) were fed either RD (13.5% of kcal from fat; LAB Diet #5010) or HFD (60% of kcal from fat; Research Diets #D12492) *ad libitum* for a period of 14–16 weeks (Supplementary Fig. 1a). For glucose tolerance test (GTT), mice were fasted for 16 hours and then glucose was administered intraperitoneally (2 g/kg of body weight). Blood glucose was measured at 0, 15, 30, 60, 90, and 120 min using a glucometer (Accu-Chek®, Roche diagnostics).

### Animal studies

For CDI, freshly prepared cefoperazone (Sigma) was administered at a concentration of 0.5 g/L in drinking water. Cefoperazone containing drinking water was changed every other day for 5 days (days −7 to −2) as previously described.^48^ Mice were then given regular drinking water for 2 days (days −2 to 0). On day 0, mice were challenged with ~10^3^ VPI 10463 spores in 100 µL sterile DPBS by oral gavage (Fig. 1a). Sham mice received 100 µL DPBS. A subset of mice were treated with oral vancomycin (50 mg/kg/day; Sigma) on day 3, 4, and 5 after infection (Fig. 3a). After infection, all mice were single-caged to prevent transfer of disease between mice and allow for appropriate diarrhea scoring. Animals were monitored daily for weight loss and diarrhea. Diarrhea was scored from zero to three based on stool consistency (0 = Normal formed stool pellet; 1 = Soft discolored stool; 2 = watery stool/wet stained tail; 3 = mucus discharge/no stool with previous episode of diarrhea).

For FXR agonist studies, *C. difficile*-infected HFD-fed mice were given either vehicle control (10% sulfobutylether β-cyclodextrin; SBE-β-CD MedChemExpress, Cat# HY-17031) or OCA (10 mg/kg; MedChemExpress, Cat# HY-12222; dissolved in 10% SBE-β-CD) every 24 h by oro-gastric gavage for a period of 10 days. To allow time for the *C. difficile* spores to reach the distal GI tract and germinate, the first dose of OCA was given 12 hours after spore inoculation (Fig. 7a).

### *Estimation of C. difficile* spore germination, outgrowth, and in vivo burden

Purified *C. difficile* spores were suspended in deoxygenated ultrapure water (10^6^ spores/ml) and heated at 65 °C for 20 min to eliminate vegetative cells. Diluted spores (10^4^ spores) were mixed with cecal contents (100 mg/mL H_2_O) and incubated for 6 h (for germination assay) and 24 h (for outgrowth assay) at 37 °C in an anaerobic chamber. Bacterial enumeration was performed by plating the mixture anaerobically on selective medium (CCFA-HT plates) in order to determine the total amount of vegetative cells and spores. A portion of spore-cecal content mixture was heat-treated at 65 °C for 20 min and plated to estimate spores. Percent germination was calculated as X = (T_*t*_ − S_*t*_)/(T_*c*_ − S_*c*_) × 100, where T_*t*_ is the total colony-forming units (CFUs) of test samples (spores inoculated in cecal contents) at 6 h, S_*t*_ is the spore CFU of test samples after heat inactivation, T_*c*_ is the total CFU of control samples (spores inoculated in brain heart infusion media supplemented with taurocholate) at 6 h, and S_*c*_ is the spore CFU of controls samples after heat inactivation. In vivo *C. difficile* burden was determined by plating 1:10 dilutions of cecal contents on CCFA-HT plates. CFU was estimated after anaerobic incubation at 37 °C for 24 h. Toxin titers were determined using the *C. difficile* TOXA/B ELISA kit from TechLab.

### Histopathology of cecal sections

Collected cecal tissue samples were fixed in Bouin’s solution (Sigma) overnight. Samples were washed and dehydrated in 70% ethanol prior to paraffin embedding. Four-micron sections were stained with hematoxylin and eosin (H&E) and scored for inflammatory cell infiltration, edema, and epithelial disruption. A score of 0 to 4, denoting increasingly severe abnormality, was assigned for each of these parameters.

### Cell enumerations by flow cytometry

Peripheral blood was obtained by intra-cardiac puncture and collected in ethylenediaminetetraacetic acid microtainers (BD Biosciences, CA, USA). Lamina propria cells were isolated as previously described,^49^ and in Supplementary methods. Infiltrating innate cells after CDI were analyzed by flow cytometry.

### Metagenomic shotgun sequencing and data analysis

DNA was extracted from cecal contents using PowerFecal DNA isolation kit (MO BIO, CA, USA). Details of library preparation, sequencing, taxonomic assignment of DNA reads and metagenomic analysis are described in Supplementary methods.

### Analysis of cecal bile acids and plasma C4 by LC-MS

Quantitative analyses of BA in cecal contents and C4 in plasma was carried out using a Waters Quattro TQ-XS triple quadruple mass spectrometer interfaced with Aquity UPLC system (Milford, MA). Cecal contents were homogenized and sonicated sequentially with 80% Methanol/Water and Chloroform/Methanol (2:1, v/v) and the supernatants were air-dried. For BA analysis, 1/20th of the dried extract was taken and 15 isotope-labeled internal standards were added prior to C18 solid phase cartridge extraction and sample clean up. Quantification of BAs was based on a stable-isotope dilution mass spectrometry method. Calibration curves were built with 23 of the BA reference compounds and detected in single ion recording (SIR) mode on mass spectrometer. Levels of individual and total BAs were normalized to cecal content weight. Plasma 7α-hydroxy-4-cholesten-3-one (C4) concentration was determined by a validated stable-isotope dilution tandem MS method using deuterium-labeled 7α-hydroxy-4-cholesten-3-one ([^2^H_7_–25,26,26,26,27,27,27]-7α-hydroxy-4-cholesten-3-one; D_7_-C4) as the internal standard. Calibration curve was constructed with C4, the reference compound and its D_7_-C4 internal standard and detected by multiple reactions monitoring (MRM) function on mass spectrometer.

### Statistical analysis

All statistical analyses were performed using GraphPad Prism 5.0 software (GraphPad software Corporation, Inc, CA, USA). For comparison of groups, a Student’s *t*-test or ANOVA with Bonferroni correction was used. A *p* value below 0.05 was considered significant.

## Supplementary information

Supplementary Methods
