# Supplementary material for: Obeticholic acid ameliorates severity of Clostridioides difficile infection in high fat diet-induced obese mice
Source: Mucosal Immunol. 2020 Aug 18;14(2):500–10. doi: 10.1038/s41385-020-00338-7 (PMC7889747; doi:10.1038/s41385-020-00338-7)
Supplement: Supplementary file 1 — Supplementary Methods [file 41385_2020_338_MOESM1_ESM.doc]

# Supplementary Methods

**Cell preparation and flow cytometry:** Lamina propria cells were isolated and enumerated as previously described^1^. In brief, ~2cm sections of cecal tissue was excised and cleaned by gentle shaking in buffer A [HBSS with 5% FCS and 25mM HEPES (Sigma)]. Clean tissue was transferred to HBSS buffer contain 2mM EDTA (Sigma) and 25mM HEPES and vortexed vigorously to remove mucus layer. Resulting tissue were mechanically minced and digested enzymatically in Iscove’s media containing liberase TL (Roche; 0.17 mg/ml) and DNase (Sigma; 30µg/ml) for 60min at 37°C shaking water bath. Enzymatic digestion was terminated by adding 2 volumes of buffer A and removed by centrifugation at 1500rpm for 6min. Resulting pellet was suspended in Buffer A and passed through 40µm cell strainer (Fisher scientific) to obtain single cell suspension. Final cell suspension was prepared in ice cold FACS buffer and cell number was determined using automated cell counter (BioRad).

Approximately 10^6^ cells per 100µL of PBS were initially stained with fixable live dead dye (Life technologies) for 15min at 4°C in a 96 well plate. Cells were washed twice and blocked with unlabeled anti-CD16/32 (Fc-block; BD Biosciences). After blocking, cells were stained with pre-titrated fluorescently labeled antibodies (**Supplementary Table 1**) in FACS buffer (2% fetal bovine serum in PBS) for 30min at 4°C. Cells were washed twice to remove unbound antibodies and fixed overnight in fixing buffer (BD Biosciences). Cells were then washed and suspended in FACS buffer. Flow cytometry was performed on a BD LSR FortessaTM (BD Biosciences) cytometer equipped with FACS-Diva (BD Biosciences) software and analyzed using FlowJo (Treestar). Gating was performed based on fluorescence-minus one (FMO) controls. Live cells were identified as leukocytes: CD45^+^; neutrophils: CD45^+^CD11b^+^Ly6G^+^; monocytes: CD45^+^CD11b^+^Ly6G^-^Ly6C^hi^; and eosinophils: CD45^+^CD11b^+^Ly6G^low^SiglecF^+^.

**Shotgun metagenome sequencing:** DNA was extracted from approximately 0.1 gm of stool using the PowerFecal® DNA isolation kit by MO BIO (MO Bio Laboratories, Carlsbad, CA) per manufacturer recommendations. DNA samples were diluted to approximately 200 ng/mL and 1 ng of Salinibacter ruber genomic DNA was added to a final concentration of 1.4 ng/mL as an internal standard. Sequencing libraries were generated from microbial DNA using the Nextera XT protocol (Illumina, San Diego, CA). Sequencing was performed on an Illumina NextSeq500 machine using 150-bp DNA paired end reads to a depth of approximately 4 G base pairs per sample. Raw sequence data was de-multiplexed and converted to fasta format and subjected to downstream analysis.

**Taxonomic assignment of DNA reads:** Paired-end sequencing reads from each sample were aligned with Kraken (version 2.0) against a custom genome database consisting of the human genome and approximately 40,054 bacterial, fungal, viral and parasitic genomes^2^. The database was derived from all complete genomes plus chromosomal and contig assemblies of bacteria, fungi, and viruses in the RefSeq genome database (<ftp://ftp.ncbi.nlm.nih.gov/genomes/refseq/>, accessed 11/27/2017) as well as the human genome database (GR38Ch;ftp://ftp.ncbi.nlm.nih.gov/genomes/ refseq/vertebrate_mammalian/Homo_sapiens/latest_assembly_versions/). Manual curation was used to add additional Bacteroides, Parabacteroides, and Clostridia genomes including draft genomes from NCBI Assemblies (https://www.ncbi.nlm.nih.gov/assembly) and PATRIC (<https://www.patricbrc.org/view/>

Taxonomy/2#view_tab=genomes). Additional fungal and viral genome sequences were recovered from the above two resources and dedicated viral (https://www.viprbrc.org/brc/home.spg?decorator=vipr, http://www.virusite.org/) and fungal (http://fungidb.org/fungidb/) databases. Reads were assigned using Kraken (version 2), which uses a k-mer based exact matching algorithm to assign reads to the lowest unambiguous taxonomic level. Taxonomic count data was then normalized to yield the same number of assigned counts per sample by rarefaction using the Vegan package in R^3^. Species or genera that contributed less than 0.01% of overall mapped reads or were present in less than 10% of the samples were then removed.

**Metagenomic Analysis:** Comparison of the overall microbiome composition between mouse groups was performed by multi-response permutation procedure (MRPP) using the Vegan package in R^3^. Unsupervised principal component analysis (PCA) was performed on log2-transformed taxonomic count data using the FactoMineR package in R^4^. To identify species or genera that significantly differed between mouse groups, Wilcoxon pairwise rank sum test was performed using the function ‘pairwise.wilcox.test’ in the stats package of R and the resulting p-values were adjusted for the false discovery rate (FDR) of < 0.1 using the ‘p.adjust’ function from the stats package in R. Shrinkage Linear Discriminant Analysis (SLDA) was utilized to calculate effect size, a measure of a species’ ability to distinguish mouse groups, as described previously^5^. SLDA is a variant of linear discriminant analysis used in LEfSe, a program widely used to select features from 16S sequencing projects^6^.

**Alignment of reads to Bile Salt Hydrolase and Bile Acid 7-α-dehydroxylase genes**: The peptide sequences of BSH and bile acid dehydroxylases were downloaded as FASTA files from NCBI (appended table with list of sequence in **Supplementary table 2**). Respective peptide database files were generated with the program diamond6. Sequence reads were aligned against the databases using diamond blastx using the ‘–sensitive’ setting^7^.

# Supplementary Tables

## Supplementary Table 1: List of Flow cytometry antibodies

| ***Antibody*** | ***Manufacturer*** | ***Clone*** |
| --- | --- | --- |
| Live/Dead *Fix Far Red* | ThermoFisher Scientific | N/A |
| CD45.2 - *PerCP-Cy5.5* | BD Pharmingen | 104 |
| CD11b - *BV650* | BD Biosciences | M1/70 |
| Ly6G - *BV421* | BioLegend | 1A8 |
| Ly6C - *PECy7* | eBioscience | HK1.4 |
| SiglecF - *PE-CF594* | BD Biosciences | E50-2440 |
| CD16/CD32 | eBioscience | 93 |

## Supplementary Table 2: List of NCBI Accession numbers of *bsh* and *bai* genes used to bin and identify total abundance of these genes in our data set

| ***Accession #*** | ***Protein*** | ***Organism*** |
| --- | --- | --- |
| *AAF67801.1* | Bile salt hydrolase | *Bifidobacterium longum* |
| *AAS98803.1* | Bile salt hydrolase | *Bifidobacterium animalis* |
| *ABC26911.1* | Bile salt hydrolase | *Bifidobacterium breve* |
| *ACL98173.1* | Bile salt hydrolase | *Lactobacillus acidophilus* |
| *ACL98201.1* | choloylglycine hydrolase | *Lactobacillus salivarius* |
| *ACL98203.1* | Bile salt hydrolase | *Lactobacillus salivarius* |
| *AEH93162.1* | conjugated bile salt acid hydrolase | *Listeria monocytogenes* |
| *AEU11039.1* | Bile salt hydrolase | *Lactobacillus plantarum* |
| *AJE05964.1* | choloylglycine hydrolase | *Bifidobacterium adolescentis* |
| *EFV15349.1* | choloylglycine hydrolase family Linear amide C-N hydrolase | *Lachnospiraceae bacterium 5_1_63FAA* |
| *KJJ74820.1* | choloylglycine hydrolase | *Clostridium sp. FS41* |
| *KMM44365.1* | choloylglycine hydrolase | *Cellulomonas sp. A375-1* |
| *SDM60055.1* | choloylglycine hydrolase | *Actinomyces ruminicola* |
| *SYZ33735.1* | choloylglycine hydrolase | *Propionibacterium australiense* |
| *WP_090143580.1* | choloylglycine hydrolase | *Clostridium perfringens* |
| *AAB61155.1* | 3alpha-hydroxy bile acid-CoA-ester 3-dehydrogenase 1/3 | *Clostridium scindens* |
| *CUM78994.1* | Bile acid 7-dehydroxylase 1/3 | Ruminococcus torques |
| *EDS06021.1* | Bile acid 7-dehydroxylase 1/3 | Clostridium scindens |
| *EEX21040.1* | Bile acid 7-dehydroxylase 1/3 | Blautia hansenii |
| *EGN35925.1* | bile acid 7-dehydroxylase 2 | *Lachnospiraceae bacterium 5_1_57FAA* |
| *EGN36052.1* | bile acid 7-dehydroxylase 1/3 | *Lachnospiraceae bacterium 5_1_57FAA* |
| *SCG90557.1* | Bile acid 7-dehydroxylase 1/3 | uncultured Clostridium sp. |
| *SCG99922.1* | Bile acid 7-dehydroxylase 1/3 | uncultured Blautia sp. |
| *SCH35738.1* | Bile acid 7-dehydroxylase 1/3 | uncultured Ruminococcus sp. |
| *SCJ02249.1* | Bile acid 7-dehydroxylase 1/3 | uncultured Roseburia sp. |
| *AAC45411.2* | 3-oxocholoyl-CoA 4-desaturase | Clostridium scindens |
| *AAC45417.1* | 7-beta-hydroxy-3-oxochol-24-oyl-CoA 4-desaturase | Clostridium scindens |


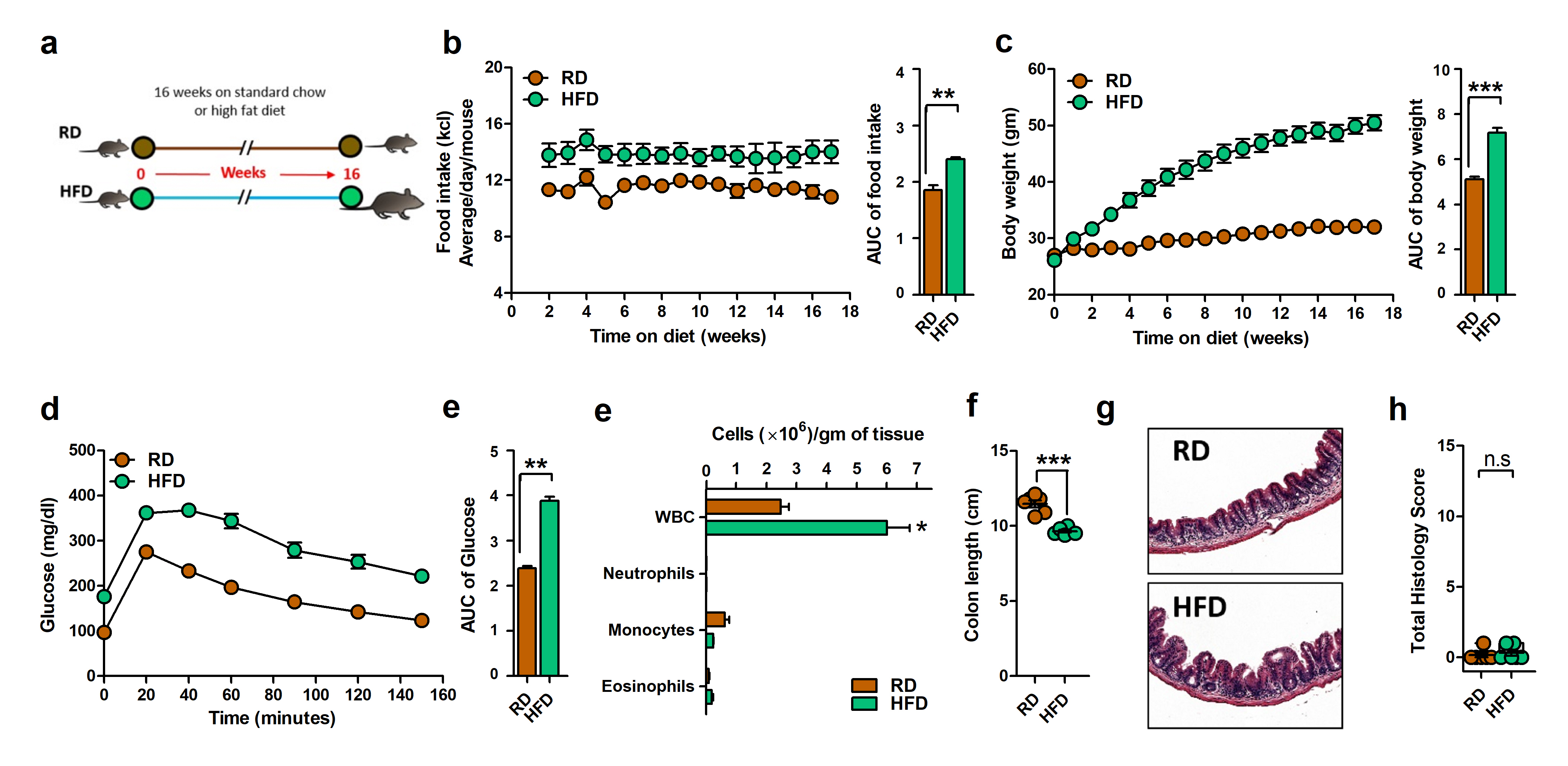


**Supplementary Figure 1.** Schematic representation of dietary modification: age-matched male C57BL/6 mice were fed with regular chow diet (RD-fed) or high fat diet (HFD-fed) for 16 weeks to establish obesity ***(a)***. Food intake ***(b)*** and weight gain ***(c)*** during dietary modification. GTT at 15 weeks ***(d)***. Number of WBCs, neutrophils, monocytes and eosinophils in cecal tissue in naive RD-fed and HFD-fed mice ***(e).*** *Data are means ± SEM. n=12 for* ***b-d****; n = 4-8 for* ***e****;* *n = 6 for* ***f*** *and* ***h****. *p < 0.05, **p < 0.01, ***p < 0.001; 2-tailed Student’s t-test*.


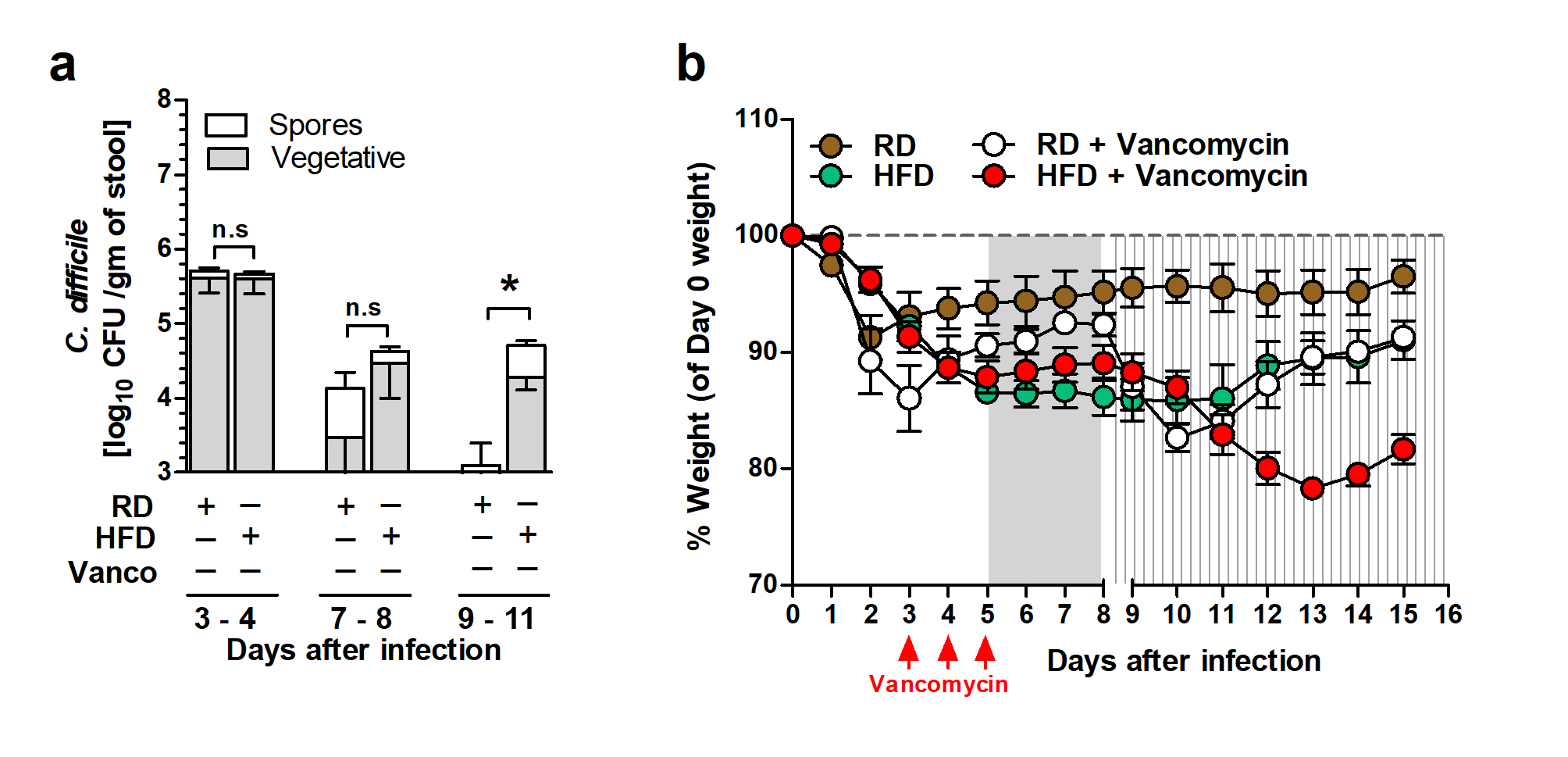


**Supplementary Figure 2.** *C. difficile* CFU in stool samples of mice that were used as controls in vancomycin treatment experiment ***(a).*** Percent body weight of mice after CDI and vancomycin treatment showing increased weight loss in HFD-fed mice during the recurrent phase (indicated by shaded lines in the background) after vancomycin treatment ***(b) .*** *Data are means ± SEM. n = 6 per group. *p < 0.05, n.s. non-significant; 2-tailed Student’s t-test.*


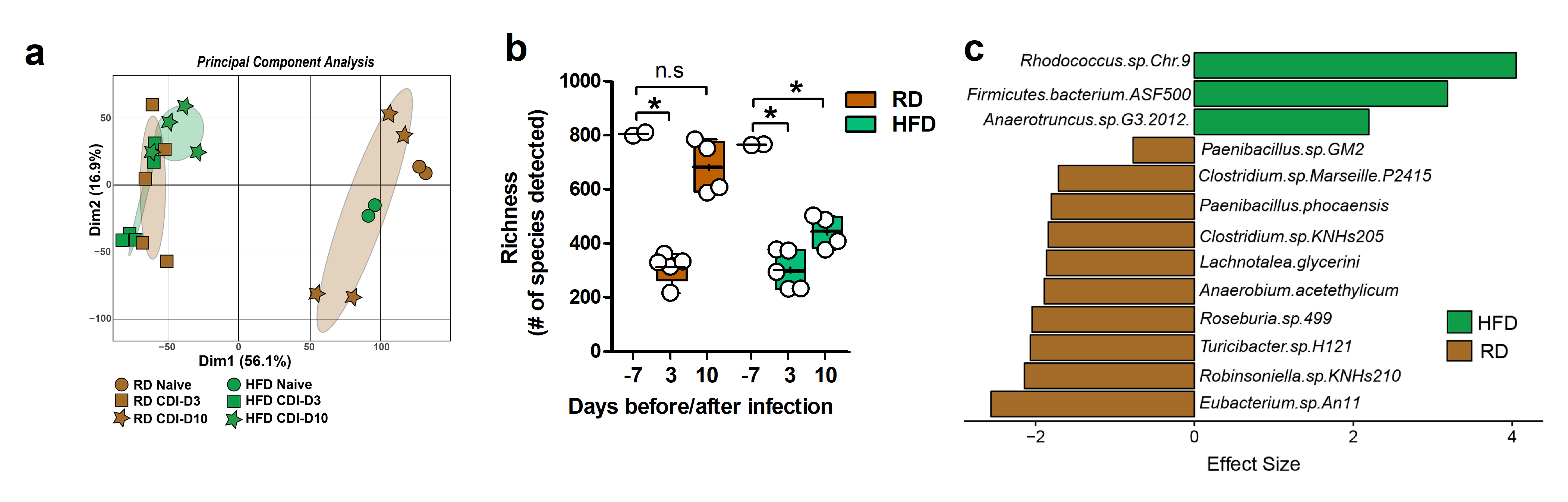


**Supplementary Figure 3.** Principal component analysis (PCA) plot of cecal microbiota profiles (at the species level) of naïve and *C. difficile-*infected (day 3 and day 10) mice. The ellipses represent 95% confidence interval around the calculated group centroid. ***(a)***. Number of species detected (species richness) ***(b)***. Shrinkage linear discriminant analysis of effect size plot of species that significantly affect the microbial community on day 3 of CDI *(Cut off p value set at 0.025 and effect size at 0.3). Data are means ± SEM. n = 2-5 per group. *p < 0.05, n.s. non-significant; 2-tailed Student’s t-test.*


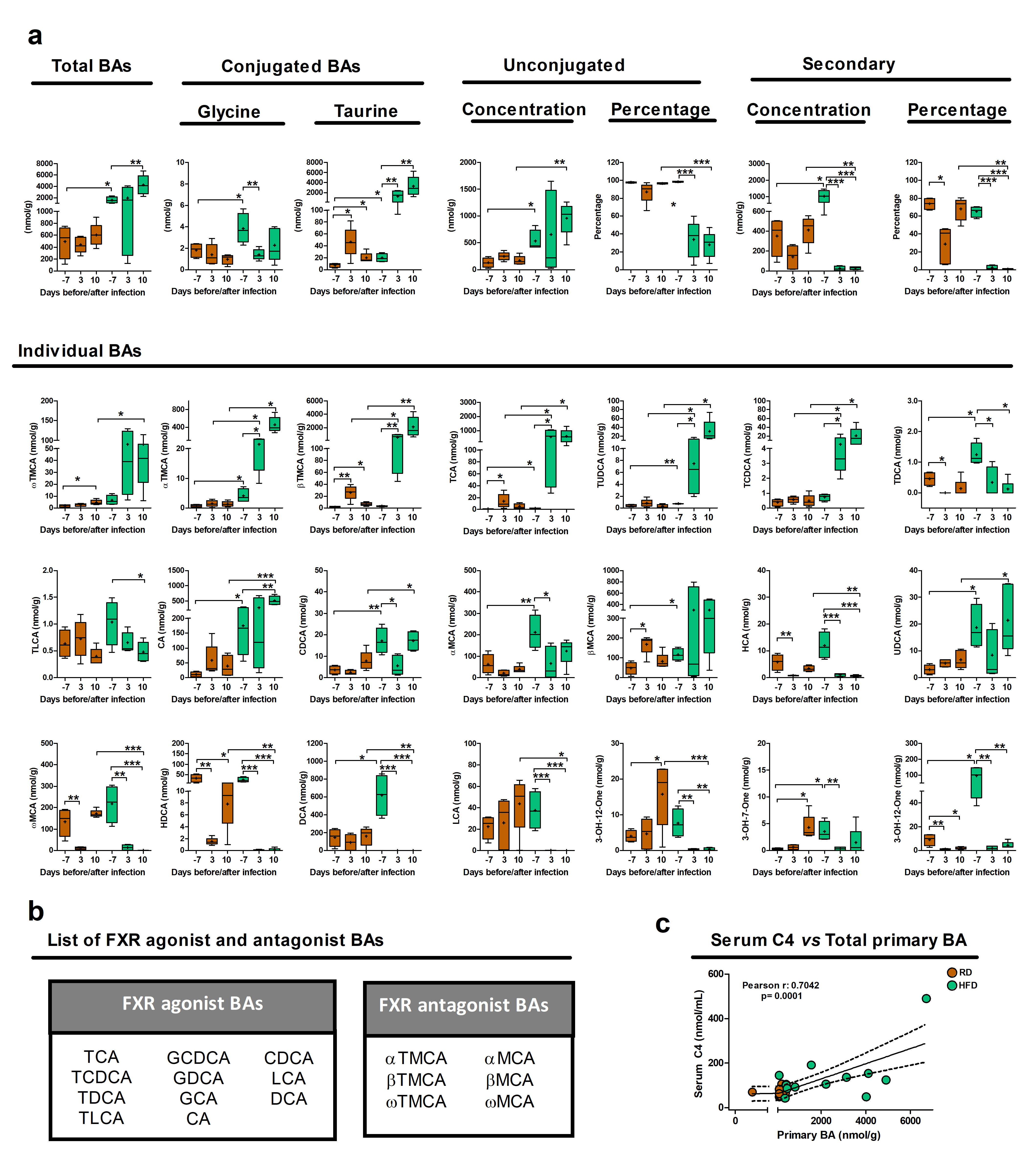


**Supplementary Figure 4.** Concentration of total, conjugated, unconjugated, secondary and individual bile acids (BAs) in cecal contents of RD-fed and HFD-fed mice before and after CDI ***(a)***. Tables show list of FXR agonist and antagonist BAs ***(b)***. Pearson correlation of serum C4 and total primary bile acids in cecal contents ***(c).*** *Data are means ± SEM. n = 4-5. *p < 0.05, **p < 0.01, ***p < 0.001, n.s. non-significant; 2-tailed Student’s t-test.*

**
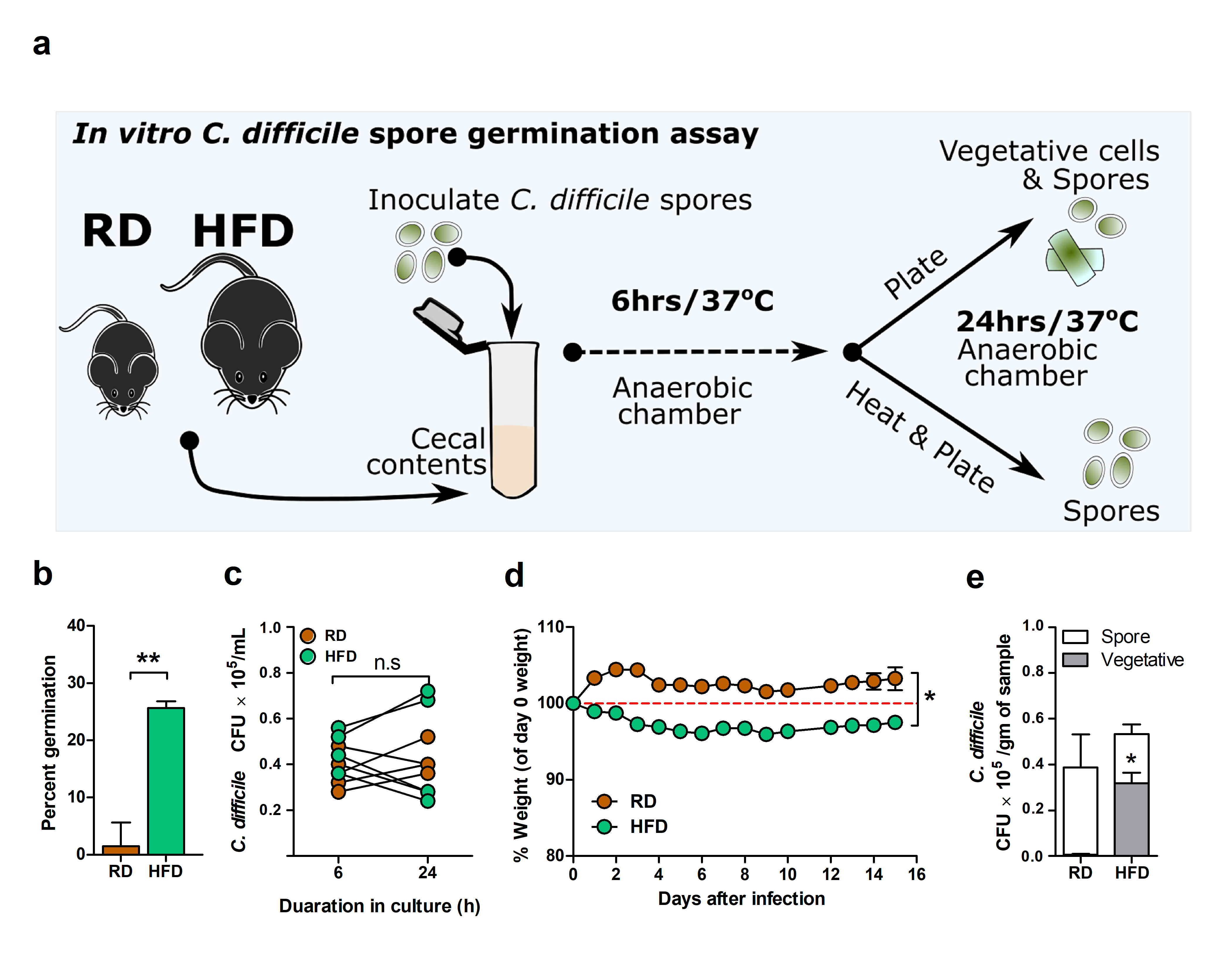
**

**Supplementary Figure 5.** Experimental plan: *In vitro* germination and outgrowth of *C. difficile* spores in cecal contents collected from RD-fed and HFD-fed mice ***(a)***. Percent germination of *C. difficile* spores inoculated in cecal contents ***(b)***. Number of C. difficile CFU at 6h and 24h after inoculation in cecal contents ***(c)***. Percent weight change of RD-fed and HFD-fed mice after *C. difficile* challenge without antibiotic pretreatment ***(d)***. *C. difficile* burden in stool samples (day 4) of mice challenged with *C. difficile* spores without antibiotic pretreatment ***(e).*** *Data are means ± SEM. n = 6-8 per group. *p < 0.05; 2-tailed Student’s t-test.*

*
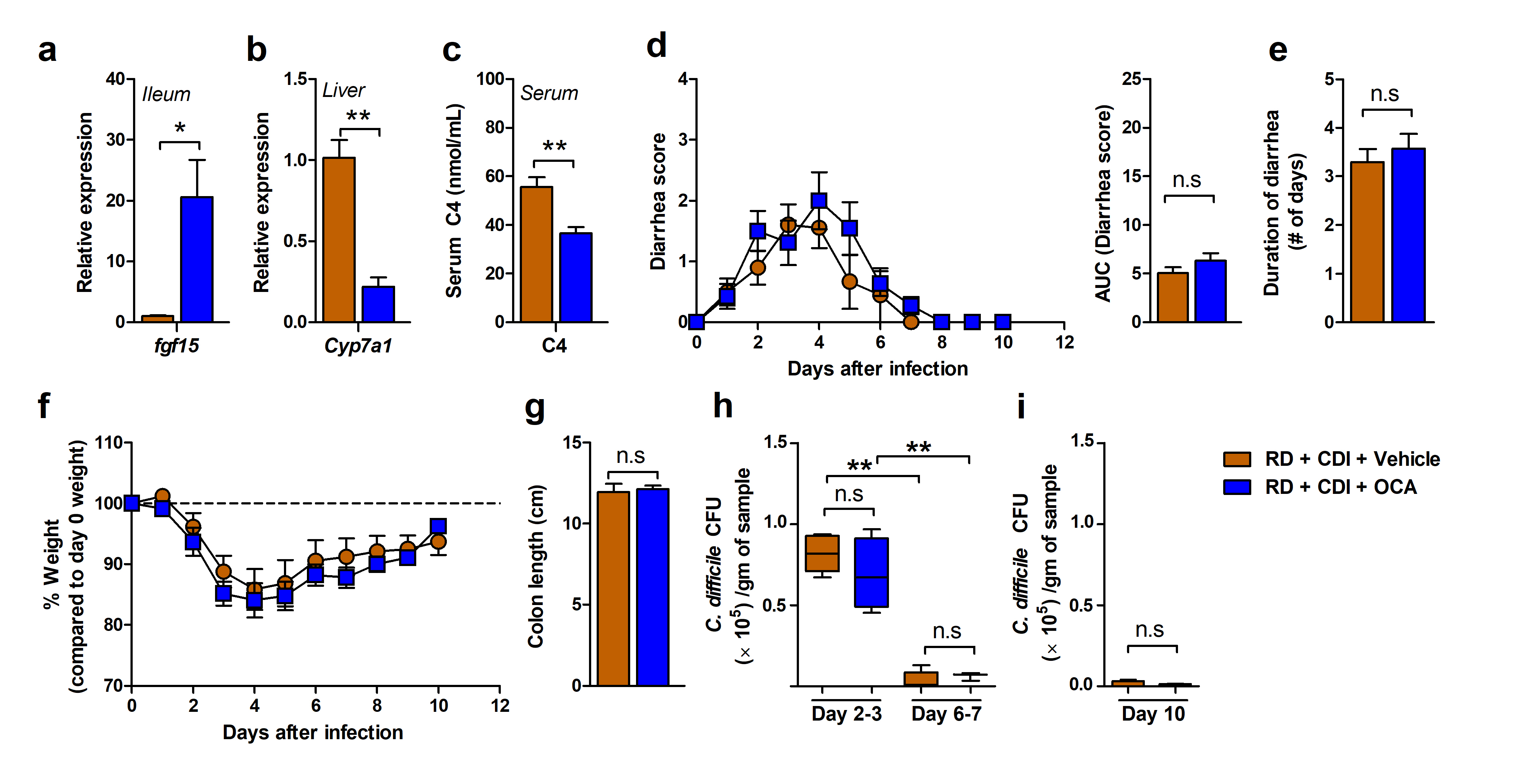
*

**Supplementary Figure 6:** mRNA expression of *fgf15* in distal ileum ***(a),*** *Cyp7a1* in liver tissue ***(b)****,* and C4 concentration in serum ***(c)*** on day 10 of CDI in RD-fed mice. Disease severity was evaluated based on diarrhea score ***(d)***, duration of diarrhea ***(e)***, and percent body weight change after CDI ***(f)****.* Extent of tissue damage was evaluated based on changes in colon length ***(g)***, *C. difficile* burden in stool samples ***(h)***, cecal contents ***(i)***. *Data are means ± SEM. n=6 for* ***a-c****; n = 9-14 for* ***d-i****; data presented in* ***d-i*** *are pooled from two independent experiments. *p < 0.05, **p < 0.01, ***p<0.001; 2-tailed Student’s t-test.*

# Supplementary References:

1. Jose S, Abhyankar MM, Mukherjee A, Xue J, Andersen H, Haslam DB *et al.* Leptin receptor q223r polymorphism influences neutrophil mobilization after Clostridium difficile infection. *Mucosal Immunol* 2018; **11**(3)**:** 947-957.

2. Wood DE, Salzberg SL. Kraken: ultrafast metagenomic sequence classification using exact alignments. *Genome Biol* 2014; **15**(3)**:** R46.

3. Ordination methods, diversity analysis and other functions for community and vegetation ecologists. *vegan: Community Ecology Package* 2015.

4. Lê S, Josse J, Husson F. FactoMineR: An R Package for Multivariate Analysis. *2008* 2008; **25**(1)**:** 18.

5. Hellmann J, Andersen H, Fei L, Linn A, Bezold R, Lake K *et al.* Microbial Shifts and Shorter Time to Bowel Resection Surgery Associated with C. difficile in Pediatric Crohn's Disease. *Inflamm Bowel Dis* 2019.

6. Segata N, Izard J, Waldron L, Gevers D, Miropolsky L, Garrett WS *et al.* Metagenomic biomarker discovery and explanation. *Genome Biol* 2011; **12**(6)**:** R60.

7. Buchfink B, Xie C, Huson DH. Fast and sensitive protein alignment using DIAMOND. *Nature methods* 2015; **12**(1)**:** 59-60.
